# Influence of nutrient signals and carbon allocation on the expression of phosphate and nitrogen transporter genes in winter wheat (*Triticum aestivum* L.) roots colonized by arbuscular mycorrhizal fungi

**DOI:** 10.1371/journal.pone.0172154

**Published:** 2017-02-16

**Authors:** Hui Tian, Xiaolei Yuan, Jianfeng Duan, Wenhu Li, Bingnian Zhai, Yajun Gao

**Affiliations:** Key Laboratory of Plant Nutrition and Agri-environment in Northwest China, Ministry of Agriculture, College of Natural Resources and Environment, Northwest A&F University, Yangling, Shaanxi, China; Estacion Experimental del Zaidin, SPAIN

## Abstract

Arbuscular mycorrhizal (AM) colonization of plant roots causes the down-regulation of expression of phosphate (Pi) or nitrogen (N) transporter genes involved in direct nutrient uptake pathways. The mechanism of this effect remains unknown. In the present study, we sought to determine whether the expression of Pi or N transporter genes in roots of winter wheat colonized by AM fungus responded to (1) Pi or N nutrient signals transferred from the AM extra-radical hyphae, or (2) carbon allocation changes in the AM association. A three-compartment culture system, comprising a root compartment (RC), a root and AM hyphae compartment (RHC), and an AM hyphae compartment (HC), was used to test whether the expression of Pi or N transporter genes responded to nutrients (Pi, NH_4_^+^ and NO_3_^-^) added only to the HC. Different AM inoculation density treatments (roots were inoculated with 0, 20, 50 and 200 g AM inoculum) and light regime treatments (6 hours light and 18 hours light) were established to test the effects of carbon allocation on the expression of Pi or N transporter genes in wheat roots. The expression of two Pi transporter genes (*TaPT4* and *TaPHT1*.*2*), five nitrate transporter genes (*TaNRT1*.*1*, *TaNRT1*.*2*, *TaNRT2*.*1*, *TaNRT2*.*2*, and *TaNRT2*.*3*), and an ammonium transporter gene (*TaAMT1*.*2*) was quantified using real-time polymerase chain reaction. The expression of *TaPT4*, *TaNRT2*.*2*, and *TaAMT1*.*2* was down-regulated by AM colonization only when roots of host plants received Pi or N nutrient signals. However, the expression of *TaPHT1*.*2*, *TaNRT2*.*1*, and *TaNRT2*.*3* was down-regulated by AM colonization, regardless of whether there was nutrient transfer from AM hyphae. The expression of *TaNRT1*.*2* was also down-regulated by AM colonization even when there was no nutrient transfer from AM hyphae. The present study showed that an increase in carbon consumption by the AM fungi did not necessarily result in greater down-regulation of expression of Pi or N transporter genes.

## Introduction

More than two-thirds of plant species can form mutualistic associations with arbuscular mycorrhizal (AM) fungi [1]. In these associations, the AM fungi depend on carbon supplied by the host plant and, in return, provide the plant with mineral nutrients from the soil [2]. AM fungi benefit the host by enhancing acquisition of phosphate (Pi), nitrogen (N), and other essential elements such as zinc and copper [3]. AM symbioses have two pathways for nutrient uptake: a direct uptake pathway from the rhizosphere by root epidermal cells and root hairs; and an indirect uptake pathway via AM fungi [4]. Studies have shown that the contribution of the AM Pi uptake pathway can be more significant than the direct Pi uptake pathway [4, 5]. [5] speculated that colonization by AM fungi may inactivate the direct Pi uptake pathway via down-regulation of the expression of Pi transporter genes in the epidermis and root hairs. The high affinity Pi transporters responsible for the direct Pi uptake pathway are often Pi-starvation inducible and are expressed in root hairs and epidermal cells [6]. Since the late 1990s, various studies have shown that colonization by AM fungi results in the down-regulation of expression of Pi-starvation inducible Pi transporter genes in *Medicago truncatula*, maize (*Zea mays* L.), and wheat (*Triticum aestivum* L.) [7, 8]. A recent study showed that the expression of some Pi-starvation inducible Pi transporters was more than 40-fold lower in AM plants compared to non-mycorrhizal plants [9]. A split-root study also found that AM colonization regulated the expression of the Pi-starvation inducible Pi transporter genes in winter wheat in a localized manner but not systemically [10]. Nevertheless, some Pi transporters in the *Pht1* gene family do not respond to AM colonization [6].

With regard to nitrogen, AM fungi can take up nitrates, ammonium, and some organic N sources [11]. As outlined above for Pi uptake, the AM uptake pathway can also contribute significantly to N nutrition in the host plant [12]. Plants have nitrate transporters (NRT) and ammonium transporters (AMT) that regulate the direct uptake of N from soil, because both nitrate and ammonium are N sources for plants. High- and low-affinity NRTs and AMTs mediate N uptake at lower and higher nitrate or ammonium concentrations, respectively [13, 14]. The regulation of AM colonization on nitrogen transporter (NRT and AMT) genes involved in the direct N uptake pathway has also been reported. AM colonization can both down-regulate [15] and up-regulate [16] the expression of NRT genes in plant roots. A recent study showed that AM fungi regulated the expression of plant NRT and AMT genes in different manners depending on gene type and AM fungal species [10].

Little is known about the mechanism by which AM colonization regulates the expression of Pi and N transporter genes in the direct nutrient uptake pathway. [17] proposed that AM colonization regulates the expression of Pi-starvation inducible transporter genes by improving Pi nutrition in the host plant as a result of the symbiotic function, or by changing the allocation of photosynthates in plant roots after colonization by AM fungi. As both Pi addition and AM colonization can down-regulate the expression of these Pi transporter genes [18], the former explanation seems more likely. However, a recent study showed that AM colonization down-regulated the expression of Pi-starvation inducible transporter genes in winter wheat, regardless of whether Pi uptake in shoots was improved [10]. It has been known that the contribution of the AM Pi uptake pathway can be significant even without positive mycorrhizal responses in growth [4]; thus, it is possible that the down-regulation of Pi-starvation inducible transporter genes within AM colonized roots is due to the transfer of a Pi nutrient signal from the fungus to the plant, and not to a positive mycorrhizal response in Pi uptake. The allocation of photosynthetic carbon can also influence the expression of the Pi or N transporter genes. Studies have indicated that the expression of some Pi transporter genes, NRT and AMT genes, is higher under light conditions than under dark conditions. This response may due to the decreased carbon allocated to roots under dark conditions [17, 19]. In AM symbiosis, the fungi are completely dependent on a supply of photosynthetically fixed carbon from the host plant to survive. It has been estimated that 4–20% of the carbon fixed by the host plant is allocated to the AM fungal symbiont [20]. Thus, these observations support the hypothesis of [17] that changes in photosynthate allocation may contribute to the regulation of Pi or N transporter gene expression after AM colonization. Nevertheless, this hypothesis still requires further testing to confirm its validity.

Winter wheat (*Triticum aestivum*) is one of the most important food crops in the world; however, the molecular aspects of its N uptake mechanisms are poorly understood. This is mainly because wheat is a hexaploid plant with a huge genome, a high number of repetitive DNA sequences, a low regeneration ability and a long life cycle [21]. Compared with model plants, fewer numbers of Pi or N transporter genes in wheat can be found in the NCBI database, and there is a very limited number of studies that have focused on the expression or function of these genes. Two Pi starvation-inducible Pi transporter genes (*TaPH1*.*2* and *TaPT4*), two putative low-affinity NRT gene (*TaNRT1*.*1* and *TaNRT1*.*2*) and three putative high-affinity NRT genes (*TaNRT2*.*1*, *TaNRT2*.*2* and *TaNRT2*.*3*) were found be down-regulated by AM colonization in our previous study [10]. Thus, these genes were selected in the present study to investigate the mechanisms of the down-regulation of AM colonization on Pi/N transporter genes that are involved in the direct nutrient uptake pathway in winter wheat. We tested the hypotheses that (1) Pi/N nutrient signals transferred by AM extra-radical hyphae are one of the reasons that AM colonization down-regulated the selected genes; and that (2) carbon allocation changes after the formation of the AM association are another possible explanation for the reduced expression of the selected genes in roots of winter wheat.

## Methods and materials

### Plant materials and growth conditions

#### Experiment I

**Fig 1:**
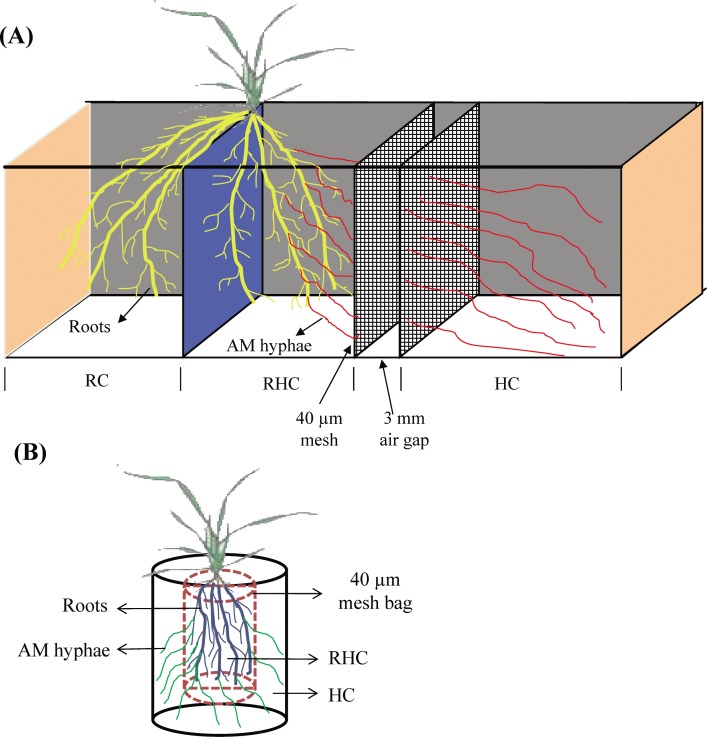
Culture systems used in experiment I (A) and II (B). RC, root compartment; RHC, root and hyphal compartment; HC, hyphal compartment.

To estimate the effect of Pi or N nutrient signals transferred by AM extra-radical hyphae on the expression of Pi or N transporter genes involved in the direct nutrient uptake pathway in AM-wheat association, a three-compartment culture system was used in experiment I (Fig 1A). This culture system had a root compartment (RC) that included only roots, a root and AM hyphae compartment (RHC) that included both roots and AM hyphae, and an AM hyphae compartment (HC) that included only AM hyphae. Two 40-μm meshes were present in the RHC and HC to prevent roots entering the HC; the two meshes were separated by a 3-mm wide air gap to prevent nutrient transfer between the RHC and HC (Fig 1A). Approximately 800 g of quartz sand, which had been washed in distilled water, was placed into each compartment of the culture system. Five treatments were set up: (1) NM; (2) *F*.*m*-nutrient; (3) *F*.*m*+NO_3_^-^; (4) *F*.*m*+NH_4_^+^; and (5) *F*.*m*+Pi (Table 1). To test whether nutrient transfer could occur from the HC to RHC via the meshes, K^15^NO_3_ was added to the HC in the NM treatment. The inoculation of RHCs with *Funneliformis mosseae* (T.H. Nicolson & Gerd.) C. Walker & A. Schüßler (BGC-NM03D) was carried out by first isolating *F*. *mosseae* spores from 150 g AM inoculum by the wet sieving method [22]; the spores were mixed with the quartz sand in the RHCs. Seeds of winter wheat (*T*. *aestivum*, cultivar Xiaoyan 22) were surface-sterilized with 1% sodium hypochlorite for 10 min, rinsed with deionized water, and then pre-germinated on moist filter paper in the dark at 25°C for 24 h. Pre-germinated seeds were then placed on 1/10 Hoagland nutrient solution for 1 week at 25°C in a greenhouse. One-week-old wheat plants were used in the experiment: each plant was placed in the culture system and roots were distributed equally between the RC and RHC. To maintain the growth of wheat plants, Hoagland nutrient solution was provided once per week to the RC in all treatments. In this experiment, there was almost no N and Pi transfer in the AM extra-radical hyphae in the *F*.*m*-nutrient treatment because only distilled-water was added to the RHC and HC. In the treatments *F*.*m*+NO_3_^-^, *F*.*m*+NH_4_^+^, and *F*.*m*+Pi, N or Pi nutrients received by the roots in the RHC could only originate from AM extra-radical hyphae as only distilled water was added to the RHC. There were four replicates for each treatment.

**Table 1:** The designs of experiment I and II.

	Treatments		Design
Experiment I	NM		No compartment was inoculated with AM fungus
	*F*.*m*-nutrient		AM fungus *Funneliformis mosseae* (BGC-NM03D) was used to inoculate only the root and AM hyphae compartment (RHC), and distilled water was added to the AM hyphae compartment (HC)
	*F*.*m*+NO_3_^-^		The RHC was inoculated with *F*. *mosseae* and ^15^NO_3_^-^ (10 mM K^15^NO_3_) was added to the HC
	*F*.*m*+NH_4_^+^		The RHC was inoculated with *F*. *mosseae* and ^15^NH_4_^+^ (10 mM (^15^NH_4_)_2_SO_4_) was added to the HC
	*F*.*m*+Pi		The RHC was inoculated with *F*. *mosseae* and Pi (1 mM KH_2_PO_4_) was added to the HC
Experiment II	18-h light	NM	wheat plants were not inoculated with AM fungus
		*F*.*m* 20	Roots were inoculated with 20 g of *F*. *mosseae*
		*F*.*m* 50	Roots were inoculated with 50 g of *F*. *mosseae*
		*F*.*m* 200	Roots were inoculated with 200 g of *F*. *mosseae*
	6-h light	NM	wheat plants were not inoculated with AM fungus
		*F*.*m* 20	Roots were inoculated with 20 g of *F*. *mosseae*
		*F*.*m* 50	Roots were inoculated with 50 g of *F*. *mosseae*
		*F*.*m* 200	Roots were inoculated with 200 g of *F*. *mosseae*

#### Experiment II

A second experiment (experiment II) was established to investigate the influence of carbon allocation on the expression of Pi or N transporter genes involved in the direct nutrient uptake pathway in the AM-wheat association. To determine the effects of different carbon allocations to AM fungi or plant roots, we compared treatments with different amounts of AM inoculant and treatments grown under different light conditions. We assumed that the greater the amount of AM fungus in the roots, the greater the carbon consumption by the AM fungus. Four AM colonization treatments (1) NM; (2) *F*.*m* 20; (3) *F*.*m* 50, and (4) *F*.*m* 200 were established (Table 1). Two light regimes were also applied: (1) 18-h light, a long light exposure in which the wheat plants received 18 hours light; and (2) 6-h light, a short light exposure in which the wheat plants were covered with a black plastic bag for 18 hours and exposed to light for 6 hours. To estimate the amount of new growth in AM extra-radical hyphae, a two-compartment culture system, with RHC and HC, was used for this experiment (Fig 1B). The HC was constructed using a 40-μm mesh bag with a volume of 200 ml. To inoculate the roots, the AM inoculum (which included AM spores, hyphae, and colonized plant root) was mixed with 200 g pasteurized soil-sand mix (containing 25% soil and 75% quartz sand) in the mesh bag. Pre-germinated wheat seeds were planted in the mesh bag, and pots were watered with tap water when needed.

### Harvest

In experiment I, wheat plants were harvested after 3 months of growth. Shoots were cut, oven-dried at 75°C for 72 h, and then weighed. Dry shoots were ground to powder using a blender (JFS-13A, China) and used to determine total N content by the Kjeldahl method [23] and Pi content as described in [24]. Shoot ^15^N content was measured using a stable isotope mass spectrometer (MAT253, Thermo Scientific, USA), and shoot ^15^N uptake was calculated using Eq (1).

$$Shoot\;{}^{15}N\;uptake\;(mg\;pot^{\text{-}1})=shoot\;{}^{15}N\;atom\;percent\;excess\;(\%)\times shoot\;N\;content\;(\%)\times shoot\;dry\;weight\;(mg)\times 10^{\text{-}4}$$ (1)

Sand in the RHCs and HCs was collected and air-dried. Approximately 5 g air-dried sand was used for measurement of AM extra-radical hyphal length density (HLD) using the method of [25]. Roots collected from the RHCs were washed with distilled water and divided into two parts: one part was stored in liquid nitrogen for RNA extraction, and the other was stored at -20°C until used for measurement of AM colonization rate.

In experiment II, the photosynthetic parameters of the wheat plants were measured using a LI-6400XT Photosynthesis System (LI-COR, Lincoln, NE, USA). Wheat plants were also harvested after 3 months of growth. Shoots were cut, oven-dried at 75°C for 72 h, and then weighed. Dry shoots were ground into powder, and total N and Pi contents were measured as described above. To determine the amount of photosynthetic carbon in the shoots of the plants, shoot reducing sugar content, starch content, and total soluble sugar content were measured [26]. Roots collected from pots were washed with distilled water, and fresh weights were measured. Root samples were divided into three parts: one part was oven-dried to determine water content for calculating root dry weight; one part was stored in liquid nitrogen for later RNA extraction; and the third part was stored at -20°C for later measurement of AM colonization rate. Soil in HCs was collected and air-dried for measurement of AM extra-radical HLDs as described above.

### Quantification of root colonization by AM fungi

Roots were cut into 1-cm long segments and stained with 0.05% (w/v) Trypan blue [27], and the AM colonization rate was quantified using the magnified intersection method [28].

### RNA extraction and reverse transcription

Total RNA was extracted from 80 mg of wheat root tissues using Trizol Reagent (TransGen Biotech, China) according to the manufacturer’s instructions. Potential genomic DNA contamination was eliminated by treatment with DNase I (TransGen Biotech, China). Approximately 1 mg total RNA was used as a template for first-strand cDNA synthesis using EasyScript™ First-Strand cDNA Synthesis SuperMix (TransGen Biotech, China) according to the manufacturer's instructions. Oligo(dT)18 primers supplied with the kit were used for reverse transcription. The cDNA was stored at -20°C until analysis.

### Real-time PCR analysis of Pi and N transporter genes

Two Pi transporter genes (*TaPT4* (AK333026) and *TaPHT1*.*2* (AJ344240)), five nitrate transporter genes (*TaNRT1*.*1*(AY587265), *TaNRT1*.*2* (AY587264), *TaNRT2*.*1*(AF288688), *TaNRT2*.*2* (AF332214), *TaNRT2*.*3* (AY053452)) and an ammonium transporter gene (*TaAMT1*.*2*) (AY525638) were selected because they are all involved in the direct nutrients uptake pathway and have been reported to be down-regulated by AM colonization in roots of winter wheat [10]. For real-time PCR, 1 ml of first strand cDNA was mixed with TransStart™ Top Green qPCR superMix (TransGen Biotech, China) according to the manufacturer's instructions. Real-time PCR was conducted using the iCycler iQ5 thermal cycler (BIO-RAD, USA). Gene specific primers and the thermal cycling conditions were as described in [10]. Four biological replicates for each treatment were performed. Wheat *GAPDH* (glyceraldehyde-3-phosphate dehydrogenase) was selected as the internal control [10]. The average threshold cycle (CT) values obtained for each sample were normalized against *GAPDH*. The relative expression ratio of the gene of interest was estimated as 2^-ΔCT^, where ΔCT = CT _target gene_ minus CT_GAPDH_; standard errors were computed from the average of the ΔCT values for each biological sample [29].

### Statistical analyses

The effects of the different treatments on wheat growth, physiology, AM colonization rate, and gene expression levels were analyzed using SAS9.1 software. Means were compared by least significant difference (LSD) at the 0.05 level unless stated otherwise.

## Results

### AM colonization and growth parameters of wheat plants in experiment I

In experiment I, roots of all wheat plants in the inoculation treatments were colonized by *F*. *mosseae*. The *F*.*m*-nutrient treatment had a significantly higher AM colonization rate than the *F*.*m*+NO_3_^-^ treatments, but there was no difference among the three nutrients addition treatments (Table 2). The AM extra-radical hyphae successfully extended into HCs, and the HLDs of HCs in *F*.*m*-nutrient and *F*.*m*+NO_3_^-^ treatments were higher than those in the other two treatments (Table 2). Addition of Pi to HCs significantly decreased the HLDs in RHCs compared to the *F*.*m*-nutrient treatment (Table 2). Inoculation of AM fungus and nutrients addition did not influence the shoot dry weight, shoot N and Pi uptake of wheat plants compared to the NM treatment (Table 2). Accumulation of ^15^N in the shoot of wheat plants was observed in the *F*.*m*+NO_3_^-^ and *F*.*m*+NH_4_^+^ treatments but not the NM treatment (Table 2) suggesting that NO_3_^-^ or NH_4_^+^ in HCs were transferred by AM extra-radical hyphae to the shoots of wheat plants, and that there was no N communication between RHCs and HCs.

**Table 2:** AM colonization, shoot dry weight, shoot N uptake, Pi uptake and AM hyphal length density in experiment I.

Treatment	AM colonization (%)	Shoot dry weight (g pot^-1^)	Shoot N uptake (mg pot^-1^)	Shoot ^15^N uptake (mg pot^-1^)	Shoot Pi uptake (mg pot^-1^)	AM hyphal length density in RHCs (m g^-1^ sand)	AM Hyphal length density in HCs (m g^-1^ sand)
NM	-	0.55 ± 0.04 a	4.82 ± 0.12 a	-	0.45 ± 0.07 a	-	-
*F*.*m*-nutrient	46.9 ± 8.7 a	0.56 ± 0.11 a	4.18 ± 0.60 a	-	0.39 ± 0.07 a	5.92 ± 0.33 a	5.62 ± 0.69 a
*F*.*m*+NO_3_^-^	21.8 ± 5.9 b	0.68 ± 0.06 a	5.69 ± 0.72 a	1.77 ± 0.22 a	0.42 ± 0.06 a	5.68 ± 0.27 ab	5.46 ± 0.22 a
*F*.*m*+NH_4_^+^	27.2 ± 8.9 ab	0.73 ± 0.04 a	5.78 ± 0.57 a	1.84 ± 0.20 a	0.34 ± 0.04 a	5.65 ± 0.28 ab	4.00 ± 0.44 b
*F*.*m*+Pi	32.9 ± 6.2 ab	0.61 ± 0.08 a	4.22 ± 0.40 a	-	0.40 ± 0.03 a	4.92 ± 0.31 b	3.50 ± 0.26 b

NM, non-mycorrhizal control; *F*.*m*-nutrient, wheat roots in RHC inoculated with the AM fungus *Funneliformis mosseae*, but no nutrients were added to HC; *F*.*m* +NO_3_^-^, +NH_4_^+^, and +Pi, wheat roots in RHC inoculated with the AM fungus *F*. *mosseae*, and ^15^NO_3_^-^ (10 mM K^15^NO_3_), ^15^NH_4_^+^ (10 Mm (^15^NH_4_)_2_SO_4_), or Pi (1 mM KH_2_PO_4_) was added to HC, respectively. Values are means and standard errors (n = 4). The same letter in a column indicates no significant difference among treatments.

### Influence of Pi and N nutrients on the expression of Pi and N transporter genes

**Fig 2:**
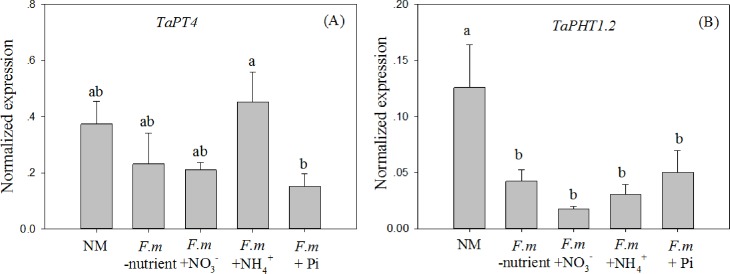
Relative expression of Pi transporter genes *TaPT4* (A) and *TaPHT1*.*2* (B) in experiment I. NM, non-mycorrhizal control; *F*.*m*-nutrient, wheat roots in RHC inoculated with the AM fungus *Funneliformis mosseae*, but no nutrients were added to HC; *F*.*m* +NO_3_^-^, +NH_4_^+^, and +Pi, wheat roots in RHC inoculated with the AM fungus *F*. *mosseae*, and ^15^NO_3_^-^ (10 mM K^15^NO_3_), ^15^NH_4_^+^ (10 Mm (^15^NH_4_)_2_SO_4_), or Pi (1 mM KH_2_PO_4_) was added to HC, respectively. Values are means and standard errors (*n* = 4). Data with the same letter indicates no significant difference among treatments (*P* > 0.05).

The expression of *TaPT4* did not change significantly in the *F*.*m*-nutrient, *F*.*m*+NO_3_^-^_,_ and *F*.*m*+NH_4_^+^ treatments compared to the NM treatment. However, when Pi was added to HCs, the expression was down-regulated in plants colonized by AM fungi (Fig 2A). These results suggest that the effect of AM colonization on the expression of *TaPT4* may be mediated through Pi transfer from the AM extra-radical hyphae. In contrast, the expression of *TaPHT1*.*2* was down-regulated in all four AM inoculation treatments, regardless of whether N or Pi was transferred by the AM extra-radical hyphae (Fig 2B).

**Fig 3:**
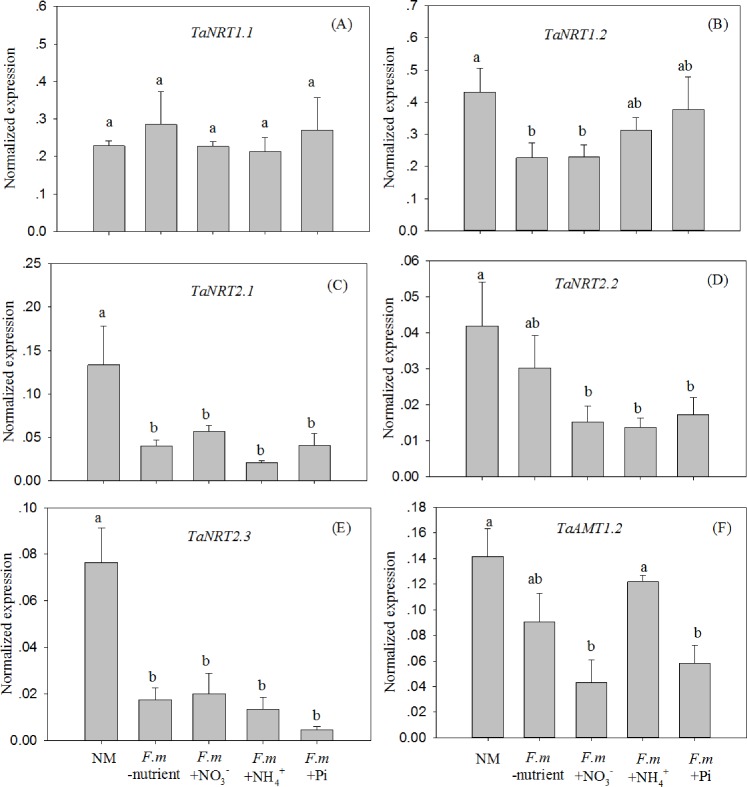
Relative expression of five nitrate transporter genes (A–E) and an ammonium transporter gene (F) in experiment I. NM, non-mycorrhizal control; *F*.*m*-nutrient, wheat roots in RHC inoculated with AM fungus *Funneliformis mosseae*, but no nutrients were added to HC; *F*.*m* +NO_3_^-^, +NH_4_^+^, and +Pi, wheat roots in RHC were inoculated with AM fungus *F*. *mosseae*, and ^15^NO_3_^-^ (10 mM K^15^NO_3_), ^15^NH_4_^+^ (10 Mm (^15^NH_4_)_2_SO_4_), or Pi (1 mM KH_2_PO_4_) was added to HC, respectively. Values are means and standard errors (*n* = 4). Data with the same letter indicates no significant difference among treatments (*P* > 0.05).

None of the four AM inoculation treatments influenced the expression of *TaNRT1*.*1* compared to the NM treatment (Fig 3A). The expression of *TaNRT1*.*2* was down-regulated in the *F*.*m*-nutrient treatment (Fig 3B), suggesting that AM colonization can down-regulate the expression even when no N or Pi transfer has occurred from the AM extra-radical hyphae. The expression of *TaNRT1*.*2* did not differ between *F*.*m*+NO_3_^-^ and *F*.*m*-nutrient treatments (Fig 3B), indicating that the down-regulation of this gene after AM colonization was not mediated by the addition of NO_3_^-^ to HCs. However, the expression of the gene was not down-regulated by AM colonization in the *F*.*m*+NH_4_^+^ and *F*.*m*+Pi treatments (Fig 3B), suggesting that the down-regulation effect might be influenced by NH_4_^+^ and Pi signals transferred from AM extra-radical hyphae. The expression of *TaNRT2*.*1* and *TaNRT2*.*3* was down-regulated by AM colonization, regardless of whether N or Pi was transferred by the AM extra-radical hyphae (Fig 3C and 3E). The levels of expression of *TaNRT2*.*2* were similar in the NM and *F*.*m*-nutrient treatments, but were higher than those in the N and Pi addition treatments (Fig 3D). This indicates that the down-regulation of *TaNRT2*.*2* expression by AM colonization largely depended on N or Pi transfer from AM extra-radical hyphae to roots of wheat plants. Similar to the results for *TaNRT2*.*2*, the down-regulation of *TaAMT1*.*2* expression following AM colonization largely depended on NO_3_^-^ and Pi rather than NH_4_^+^ transfer from AM extra-radical hyphae (Fig 3F).

### AM colonization and growth parameters of wheat plants in experiment II

The extent of AM colonization increased as the amount of AM inoculum increased in both light regime treatments (Table 3). Shoot dry weight and N and Pi uptake were improved by inoculation with AM fungus in both light regimes, but did not differ among the three AM inoculation treatments (Table 3). Compared to NM, root dry weight was decreased by the *F*.*m* 50 treatment under the 18-h light regime and was also decreased by the *F*.*m* 200 treatment under both light regimes (Table 3). A factorial ANOVA indicated that reduced light exposure decreased AM colonization, shoot dry weight, root dry weight, and shoot N and Pi uptake (*P* < 0.05). New growth of AM extra-radical hyphae was observed in HCs, and the HLD of the *F*.*m* 50 treatment was higher than that in the other two AM inoculation treatments under 18-h light conditions; HLDs in *F*.*m* 50 and *F*.*m* 200 treatments were higher than that in the *F*.*m* 20 treatment under the 6-h light conditions (Table 3).

**Table 3:** AM colonization, shoot and root dry weight, shoot N and Pi uptake of wheat plants and hyphal length density in HCs in experiment II.

Light regime	AM treatment	AM colonization (%)	Shoot dry weight (g pot^-1^)	Root dry weight (g pot^-1^)	Shoot N uptake (mg pot^-1^)	Shoot Pi uptake (mg pot^-1^)	Hyphal length density in HCs (m g^-1^ soil)
18-h light	NM	-	0.56 ± 0.03 b	0.33 ± 0.01 a	15.8 ± 0.79 b	1.09 ± 0.07 b	-
	F.m 20	28.0 ± 3.6 b	0.68 ± 0.03 a	0.29 ± 0.02 ab	26.1 ± 1.45 a	2.40 ± 0.18 a	0.54 ± 0.01 b
	F.m 50	37.8 ± 7.5 ab	0.66 ± 0.04 a	0.26 ± 0.02 b	24.7 ± 1.85 a	2.05 ± 0.20 a	0.98 ± 0.03 a
	F.m 200	44.8 ± 3.8 a	0.64 ± 0.03 a	0.25 ± 0.02 b	22.3 ± 1.75 a	2.13 ± 0.12 a	0.56 ± 0.06 b
6-h light	NM	-	0.44 ± 0.26 b	0.16 ± 0.02 a	12.6 ± 0.71 b	0.48 ± 0.04 b	-
	F.m 20	14.4 ± 1.8 b	0.53 ± 0.04 a	0.13 ± 0.01 ab	17.2 ± 0.93 a	1.87 ± 0.27 a	0.40 ± 0.02 b
	F.m 50	20.4 ± 3.1 ab	0.55 ± 0.01 a	0.12 ± 0.01 ab	18.8 ± 0.19 a	1.66 ± 0.07 a	0.72 ± 0.08 a
	F.m 200	26.6 ± 3.1 a	0.58 ± 0.02 a	0.11 ± 0.01 b	20.2 ± 0.75 a	2.04 ± 0.09 a	0.69 ± 0.10 a
Significance^a^ due to:							
AM treatment		*	**	**	***	***	**
Light regime		**	***	***	***	***	ns
AM treatment × light regime		ns	ns	ns	ns	*	*

NM, non-mycorrhizal control; *F*.*m* 20, 50, and 200, roots inoculated with 20 g, 50 g or 200 g inoculum of the AM fungus *Funneliformis mosseae*, respectively. 18-h light, wheat plants received 18 hours light; 6-h light, wheat plants received 6 hours light. ns: not significant

* *P* < 0.05

** *P*< 0.01

*** *P* < 0.001.

^a^ By analysis of variance. Numbers in the same column followed by a different letter are significantly different at *P* < 0.05 in both light regime treatments.

Almost all the three photosynthetic parameters, shoot reducing sugar, starch content, and total soluble sugar content of wheat plants were increased by the three AM inoculation treatments compared to the NM treatment under 18-h light and 6-h light regimes, but decreased by reduction in light exposure in general (Table 4). However, increasing the amount of AM inoculum did not necessarily cause a corresponding increase in photosynthetic parameters or shoot photosynthetic carbon under either the 18-h light or 6-h light regimes (Table 4).

**Table 4:** Photosynthetic parameters, shoot reducing sugar, starch and total soluble sugar content of wheat plants in experiment II.

Light regime	AM treatment	Photosynthetic rate (μmol CO_2_ m^-2^ s^-1^)	Transpiration rate (mmol H_2_O m^-2^ s^-1^)	Stomatal conductance (mol H_2_O m^-2^ s^-1^)	Reducing sugar content (mg g^-1^)	Starch content (mg g^-1^)	Total soluble sugar content (mg g^-1^)
light	NM	3.12 ± 0.43 c	1.31 ± 0.27 b	0.06 ± 0.01 c	2.13 ± 0.14 c	4.01 ± 0.32 c	14.1 ± 0.60 b
	F.m 20	8.07 ± 0.55 a	1.65 ± 0.17 b	0.08 ± 0.01 b	4.96 ± 0.33 ab	5.51 ± 0.09 b	21.1 ± 0.73 a
	F.m 50	6.80 ± 0.13 ab	2.74 ± 0.06 a	0.10 ± 0.01 a	4.06 ± 0.34 b	6.10 ± 0.34 b	19.5 ± 0.49 a
	F.m 200	6.57 ± 0.42 b	2.40 ± 0.16 a	0.09 ± 0.01 ab	5.48 ± 0.42 a	7.40 ± 0.44 a	15.6 ± 0.22 b
6-h light	NM	2.28 ± 0.18 c	1.16 ± 0.11 b	0.04 ± 0.01 b	0.71 ± 0.06 c	3.17 ± 0.24 d	12.6 ± 0.31 c
	F.m 20	4.81 ± 0.06 a	1.73 ± 0.23 a	0.11 ± 0.02 a	4.55 ± 0.24 a	4.16 ± 0.25 c	16.0 ± 0.79 b
	F.m 50	3.69 ± 0.27 b	1.34 ± 0.12 ab	0.05 ± 0.01 b	3.64 ± 0.47 b	5.19 ± 0.15 b	22.1 ± 0.09 a
	F.m 200	4.44 ± 0.43 ab	1.81 ± 0.16 a	0.06 ± 0.01 b	3.48 ± 0.23 b	7.12 ± 0.31 a	15.1 ± 0.80 b
Significance^a^ due to:							
AM treatment		***	***	***	***	***	***
Light regime		***	**	***	***	**	**
AM treatment × light regime		***	***	**	*	***	ns

NM, non-mycorrhizal control; *F*.*m* 20, 50, and 200, roots inoculated with 20 g, 50 g or 200 g inoculum of the AM fungus *Funneliformis mosseae*, respectively. 18-h light, wheat plants received 18 hours light; 6-h light, wheat plants received 6 hours light. ns: not significant

* *P* < 0.05

** *P*< 0.01

*** *P* < 0.001.

^a^ By analysis of variance. Numbers in the same column followed by a different letter are significantly different at *P* < 0.05 in both light regime treatments.

### Influence of carbon allocation on the expression of Pi and N transporter genes

**Fig 4:**
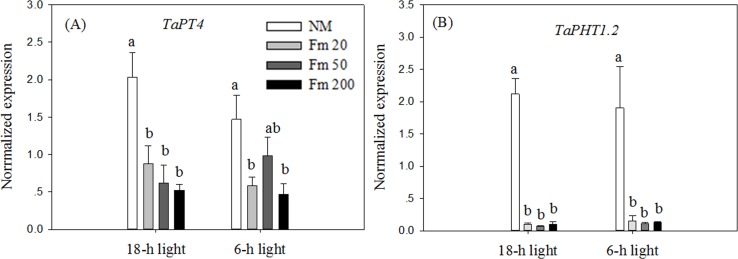
Relative expression of Pi transporter genes *TaPT4* (A) and *TaPHT1*.*2* (B) in experiment II. NM, non-mycorrhizal control; *F*.*m* 20, 50, and 200, wheat roots inoculated with 20 g, 50 g or 200 g inoculum of the AM fungus *F*. *mosseae*, respectively. Unshaded: wheat plants received 18 h light; Shaded: wheat plants received 6 h light. Values are means and standard errors (*n* = 4). Data with the same letter indicates no significant difference among AM inoculation treatments (*P* > 0.05).

The expression levels of *TaPT4* and *TaPHT1*.*2* were down-regulated by all three inoculation treatments compared to the NM treatment under both light regime treatments; the expression levels did not differ significantly among the three inoculation treatments (Fig 4A and 4B). A factorial ANOVA indicated that reduction in light exposure did not influence the expression of these two Pi transporter genes (*P* > 0.05).

**Fig 5:**
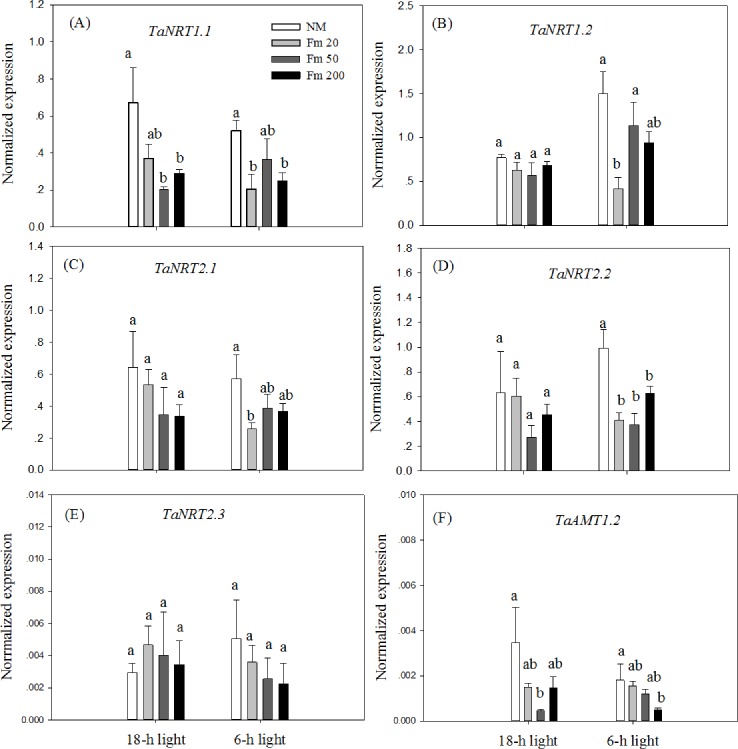
Relative expression of five nitrate transporter genes (A-E) and an ammonium transporter gene (F) in experiment II. NM, nonmycorrhizal control; *F*.*m* 20, 50, and 200, wheat roots inoculated with 20 g, 50 g, or 200 g inoculum of the AM fungus *F*. *mosseae*, respectively. Unshaded: wheat plants received 18 hours light; Shaded: wheat plants received 6 hours light. Values are means and standard errors (*n* = 4). Data with the same letter indicates no significant difference among AM inoculation treatments (*P* > 0.05).

The expression of *TaNRT1*.*1* was down-regulated by the *F*.*m* 50 and *F*.*m* 200 inoculation treatments compared to the NM treatment under the 18-h light conditions (Fig 5A). In the 6-h light treatment, the expression of *TaNRT1*.*1* was down-regulated by *F*.*m* 20 and *F*.*m* 200 inoculation treatments compared to the NM treatment (Fig 5A). The expression of *TaNRT1*.*2* was not influenced by all the AM inoculation treatments under the 18-h light conditions, and was down-regulated only by the *F*.*m* 20 treatment under the 6-h light conditions (Fig 5B). The expression of *TaNRT2*.*1* was not influenced by the AM inoculation treatment in the 18-h light treatments, and was down-regulated only by the *F*.*m* 20 treatment under the 6-h light conditions (Fig 5C). The expression of *TaNRT2*.*2* was not influenced by AM inoculation under the 18-h light conditions, but was down-regulated by AM inoculation under 6-h light conditions (Fig 5D). The expression of *TaNRT2*.*3* was not influenced by AM inoculation under either light regime (Fig 5E). The expression of *TaAMT1*.*2* was down-regulated by *F*.*m* 50 and *F*.*m* 200 treatments under 18-h light and 6-h light conditions, respectively (Fig 5F). For all NRT and AMT genes, the expression did not differ among the three AM inoculation treatments under both light regimes (Fig 5). A factorial ANOVA indicated that a reduction in light exposure did not significantly influence the expression of any N transporter gene (*P* > 0.05).

## Discussion

### Influence of N and Pi signals on the expression of Pi and N transporter genes

It has been widely reported that AM colonization down-regulates the expression of Pi or N transporter genes that are involved in the direct nutrient uptake pathway in plant roots [6, 9, 10]; however, the mechanisms of this effect remain largely unknown. It is assumed that improvement in the Pi or N status of plants after AM colonization might contribute to the down-regulation of expression of Pi or N transporter genes through a feedback regulation system [30]. However, in the present study, Pi or N transporters in roots could be down-regulated by AM colonization even when there was no increase in Pi and N accumulation (Table 2, Figs 2 and 3); a similar effect was also reported in previous studies [10, 17]. Thus, we hypothesize that the expression of Pi or N transporter genes is down-regulated when plant roots receive Pi or N signals transferred by AM hyphae, regardless of changes in shoot Pi or N. The expression of one Pi transporter gene (*TaPT4*), one NRT gene (*TaNRT2*.*2*) and one AMT gene (*TaAMT1*.*2*) was down-regulated only when Pi or N was added to the system (Figs 2 and 3). These results indicate that regulation of the expression of these genes by AM colonization largely depended on Pi or N transfer from AM extra-radical hyphae; this conclusion supports our hypothesis. Most of the Pi or N transporter genes that are involved in the direct nutrient uptake pathway are Pi or N-starvation inducible [19, 31]; this indicates that down-regulation of these genes is due to Pi or N transfer from AM extra-radical hyphae that alleviates Pi or N starvation in plant roots. Consistent with our study, the down-regulation of these genes by AM colonization was also found by [10]; however, the latter study and others also noted that AM fungal species differ in their abilities to regulate the expression of these genes [6, 9, 18]. The findings of the present study may help in part to explain this variability as it is well-known that AM fungi show functional diversity in their abilities to mediate Pi or N transfer from AM extra-radical hyphae to the host [32].

The expression of *TaPHT1*.*2*, *TaNRT2*.*1*, and *TaNRT2*.*3* was down-regulated by all the AM inoculation treatments (Figs 2 and 3), regardless of whether there was nutrient transfer from the AM hyphae. It is difficult to explain the down-regulation of these genes in the *F*.*m*-nutrient treatment, but we speculate that molecular signals produced during the formation of AM symbiosis might repress the expression of these genes. AM symbioses are initiated through a molecular dialogue that begins before physical contact. During the formation of the symbioses, AM fungi recognize signals (strigolactones) excreted from the roots of plants, and produce Myc-factors (chitin oligomers) that can be sensed by host plants [33, 34]. It was previously reported that a molecular signal (lyso-phosphatidylcholine) in AM symbiosis induces the AM-specific inducible Pi transporter genes *StPT3* and *StPT4* in potato (*Solanum tuberosum* L.) and *LePT4* tomato (*Solanum lycopersicum* L.) [35]. However, it remains unknown whether lyso-phosphatidylcholine can also influence the expression of Pi/N-starvation inducible Pi or N transporter genes in plant roots.

The expression of *TaNRT1*.*2* was significantly down-regulated by the *F*.*m*-nutrient and *F*.*m*+NO_3_^-^ treatments. The expression of this gene in the other two treatments also tended to be lower compared to that in NM although it was not statistically significant (Fig 3B). It is quite possible that the down-regulation of this gene after AM colonization was also N/Pi independent. The expression of *TaNRT1*.*1* was not affected by any of the four AM colonization treatments (Fig 3A); similar constitutive NRT genes that are not influenced by environmental factors have been reported previously [31].

### Influence of carbon allocation on the expression of Pi and N transporter genes

When plant roots are colonized by AM fungi, a portion of the photosynthetically fixed carbon is allocated to AM fungi [36]. In the present study, AM colonization was found to decrease root biomass in both light regimes (Table 3); this observation is consistent with a previous report [4]. There was also a trend toward a lower root biomass with a higher degree of AM colonization (Table 3), suggesting that higher levels of AM colonization consumed more carbon in the roots of wheat. However, it has been reported that AM colonization either has no effect or increases the growth of host plant roots [37] depending on the AM association type and environmental factors. The pattern of response may be related to increased photosynthesis to compensate for the increased carbon sink in AM symbiosis in some conditions [38]. Increased photosynthetic parameters and increased amounts of photosynthetic carbon (reducing sugar, starch, and total soluble sugar) were observed in shoots in AM treatments compared to those of the NM treatment (Table 4), although the biomass of the wheat roots still decreased after AM colonization. Wheat roots with variable degrees of AM colonization were obtained here. The highest AM colonization rate (44.8%) occurred in the *F*.*m* 200 treatment under the 18-h light conditions, and the lowest (14.4%) occurred in the *F*.*m* 20 treatment under the 6-h light conditions (Table 3). The presence of greater amounts of AM fungus in roots may result in the consumption of more carbon; previous studies reported a positive correlation between the degree of AM colonization and the concentration of the AM fatty acid biomarkers C16:1*cis*11 and C18:1*cis*11 [9]. In addition, we found more new growth of AM hyphae in the *F*.*m* 50 and *F*.*m* 200 treatments (Table 3), suggesting that more carbon was allocated to the AM fungus in these treatments. The present study found that both low and high levels of AM colonization were associated with significant down-regulation of expression of the Pi transporter genes *TaPT4* and *TaPHT1*.*2* (Fig 4A and 4B) and NRT and AMT genes (Fig 5). However, a level of AM colonization did not appear to be related to the extent of down-regulation Pi or N transporter gene expression (Figs 4 and 5). The results suggest that the regulation of expression of Pi or N transporter genes by AM colonization was independent of carbon consumption by the AM fungus in the roots of winter wheat. In addition, reduction in light exposure did not influence the expression of the Pi or N transporter genes in our study (*P* < 0.05). Contrasting conclusions have been reached regarding the effects of carbon allocation on the expression of Pi or N transporters: for example, [39] found that the expression of Pi transporters was induced by light in Pi-deficient white lupin (*Lupinus albus*) plants, whereas, [40] found that the expression of 11 Pi transporters in roots of winter wheat did not respond to variations in light treatments. The latter finding is consistent with those obtained in the present study. In *Arabidopsis*, the expression of *NRT2*.*1* and *NRT1* is induced by light [41]; this finding is contrary to our data here. A recent study showed that the expression of NRT genes did not vary between day and night in the dinoflagellate *Lingulodinium polyedrum* [42]. This is consistent with the present study and suggests that light regulation of the expression of NRT genes may vary among plant species.

## Conclusions

We conclude that the expression of one Pi-starvation inducible Pi transporter gene (*TaPHT1*.*2*) and three NRT genes (*TaNRT1*.*2*, *TaNRT2*.*1*, and *TaNRT2*.*3*) was down-regulated by AM colonization, regardless of whether there was Pi/N transfer from AM extra-radical hyphae; one Pi-starvation inducible Pi transporter gene (*TaPT4*), one NRT gene (*TaNRT2*.*2*) and one AMT gene (*TaAMT1*.*2*) were down-regulated by AM colonization only when roots of host plants received Pi/N signals. Different AM inoculation densities and light regimes did not influence the expression of almost all the Pi or N transporter genes in roots of winter wheat, suggesting that the down-regulation of the expression of Pi/N transporter genes involved in the direct uptake pathway is independent of carbon consumption of the AM symbiont.

## Supporting information

S1 Appendix Raw data for Table 2. (PDF)

S2 Appendix Raw data for Table 3. (PDF)

S3 Appendix Raw data for Table 4. (PDF)

S4 Appendix Raw data for Figs 2 and 3. (PDF)

S5 Appendix Raw data for Figs 4 and 5. (PDF)
