# Supplementary material for: Influence of nutrient signals and carbon allocation on the expression of phosphate and nitrogen transporter genes in winter wheat (Triticum aestivum L.) roots colonized by arbuscular mycorrhizal fungi
Source: PLoS One. 2017 Feb 16;12(2):e0172154. doi: 10.1371/journal.pone.0172154 (PMC5312871; doi:10.1371/journal.pone.0172154)
Supplement: S1 Appendix — (PDF) [file pone.0172154.s001.pdf]

AM colonization (%)

Treatment	repeat1	repeat2	repeat3	repeat4
NM	/	/	/	/
F.m -nutrient	49	67.3	46.32	24.8
F.m +NO_3^-	33.9	6.13	20.84	26.5
F.m +NH_4^+	34.42	40.5	1	32.78
F.m +Pi	34.33	15.6	37.3	44.5

shoot biomass(g pot-1)

Treatment	repeat1	repeat2	repeat3	repeat4
NM	0.44	0.59	0.63	0.52
F.m -nutrient	0.53	0.39	0.87	0.44
F.m +NO_3^-	0.6	0.64	0.64	0.85
F.m +NH_4^+	0.66	0.8	0.79	0.66
F.m +Pi	0.53	0.67	0.8	0.43

Shoot total N uptake(mg pot-1)

Treatment	repeat1	repeat2	repeat3	repeat4
NM	4.959141	4.57944	4.926941	
F.m -nutrient	4.242102	3.064263	5.816591	3.590175
F.m +NO_3^-	4.699841	4.669131	5.66317	7.718608
F.m +NH_4^+	6.556472	6.86848	5.285371	4.403639
F.m +Pi	4.25601	4.471121	5.0251	3.109467

Hyphal length density in RHC(m g-1 sand)

Treatment	repeat1	repeat2	repeat3	repeat4
NM	/	/	/	/
F.m -nutrient	5.32819	6.45701	5.96658	
F.m +NO_3^-	6.21495	4.92155	5.83544	5.75873
F.m +NH_4^+	5.39032	5.08972	5.75192	6.37523
F.m +Pi	5.34231	4.56105	4.23951	5.53643

Hyphal length density in HC(m g-1 sand)

Treatment	repeat1	repeat2	repeat3	repeat4
NM	/	/	/	/
F.m -nutrient	6.986644	4.185433	4.726977	6.594958
F.m +NO_3^-	5.660689	4.828388	5.522455	5.83112
F.m +NH_4^+	2.789177	4.880232	4.089452	4.221174

F.m +Pi	3.050728	3.2479	4.250687	3.466237

Shoot Pi uptake(mg pot-1)

Treatment	repeat1	repeat2	repeat3	repeat4
NM	0.375206	0.394917	0.65104	0.373824
F.m -nutrient	0.373438	0.335509	0.581728	0.280877
F.m $+NO_3^-$	0.331717	0.421166	0.355896	0.579622
F.m $+NH_4^+$	0.352682	0.4272	0.365998	0.220496
F.m +Pi	0.388231	0.459406	0.415348	0.317373

Shoot ^{15}N uptake(mg pot-1)

Treatment	repeat1	repeat2	repeat3	repeat4
NM	/	/	/	/
F.m -nutrient	/	/	/	/
F.m $+NO_3^-$	2.398561	2.451022	1.330573	1.177364
F.m $+NH_4^+$	1.073508	1.703248	2.523059	
F.m +Pi	/	/	/	/
