# Supplementary material for: Influence of nutrient signals and carbon allocation on the expression of phosphate and nitrogen transporter genes in winter wheat (Triticum aestivum L.) roots colonized by arbuscular mycorrhizal fungi
Source: PLoS One. 2017 Feb 16;12(2):e0172154. doi: 10.1371/journal.pone.0172154 (PMC5312871; doi:10.1371/journal.pone.0172154)
Supplement: S2 Appendix — (PDF) [file pone.0172154.s002.pdf]

AM colonization (%)

	Treatment	repeat1	repeat2	repeat3	repeat4
18-hour light	NM	/	/	/	/
	F.m 20	31.83	23.63	20.37	36.07
	F.m 50	26.07	25.17	43.57	56.5
	F.m 200	34.47	45.2	46.87	52.73
6-hour light	NM	/	/	/	/
	F.m 20	9.93	15.4	18.53	13.73
	F.m 50	21.13	11.53	23.7	25.27
	F.m 200	17.87	31.17	30.7	26.6

Shoot dry weight(g pot-1)

	Treatment	repeat1	repeat2	repeat3	repeat4
18-hour light	NM	0.5146	0.641	0.5579	0.5421
	F.m 20	0.7651	0.6151	0.6519	0.6767
	F.m 50	0.7457	0.7203	0.6372	0.552
	F.m 200	0.6476	0.5914	0.7225	0.5897
6-hour light	NM	0.4892	0.4565	0.4149	0.3811
	F.m 20	0.5013	0.61	0.4109	0.5823
	F.m 50	0.546	0.5643	0.5479	0.5358
	F.m 200	0.5922	0.5971	0.5213	0.6062

Root dry weight(g pot-1)

	Treatment	repeat1	repeat2	repeat3	repeat4
18-hour light	NM	0.304166	0.317972	0.352199	0.331474
	F.m 20	0.344598	0.309022	0.248471	0.266554
	F.m 50	0.302302	0.234812	0.277211	0.2426
	F.m 200	0.244377	0.249804	0.307762	0.204393
6-hour light	NM	0.227039	0.147518	0.14756	0.127464
	F.m 20	0.119555	0.110932	0.120856	0.155659
	F.m 50	0.122353	0.1209	0.136413	0.115768
	F.m 200	0.095418	0.084173	0.099333	0.122963

Shoot total N uptake(mg pot-1)

	Treatment	repeat1	repeat2	repeat3	repeat4
18-hour light	NM	15.04129	17.83592	16.17075	14.20465
	F.m 20	30.20254	25.71111	24.71989	23.55158
	F.m 50	29.3335	21.117	25.76933	22.35685
	F.m 200	20.50723	19.83021	27.47673	21.51507
6-hour light	NM	14.07872	12.78449	12.70355	10.64943
	F.m 20	16.43519	19.68564	15.32406	17.20842
	F.m 50	18.77683	18.64063	18.53533	19.37615
	F.m 200	20.53648	18.39378	19.89802	21.99392

Shoot Pi uptake(mg pot-1)

	Treatment	repeat1	repeat2	repeat3	repeat4
18-hour light	NM	1.206505	1.206808	1.053056	0.90773
	F.m 20	2.522764	2.851263	2.095302	2.119582
	F.m 50	2.479716	2.310177	1.768613	1.635704
	F.m 200	2.116145	1.901257	2.458936	2.05033
6-hour light	NM	0.510586	0.557169	0.472409	0.366632
	F.m 20	1.887662	2.379316	1.134434	2.086009
	F.m 50	1.655304	1.729877	1.803712	1.46665
	F.m 200	2.018831	2.039365	1.815635	2.272669

Hyphal length density in HC(m g-1 sand)

	Treatment	repeat1	repeat2	repeat3	repeat4
18-hour light	NM	/	/	/	/
	F.m 20	0.533994	0.506427	0.560107	0.54457
	F.m 50	0.956228	1.013004	0.975	
	F.m 200	0.493845	0.674047	0.49961	
6-hour light	NM	/	/	/	/
	F.m 20	0.373388	0.391407	0.431178	
	F.m 50	0.776256	0.556375	0.823033	
	F.m 200	0.65493	0.539777	0.885848	
