# Supplementary material for: Influence of nutrient signals and carbon allocation on the expression of phosphate and nitrogen transporter genes in winter wheat (Triticum aestivum L.) roots colonized by arbuscular mycorrhizal fungi
Source: PLoS One. 2017 Feb 16;12(2):e0172154. doi: 10.1371/journal.pone.0172154 (PMC5312871; doi:10.1371/journal.pone.0172154)
Supplement: S3 Appendix — (PDF) [file pone.0172154.s003.pdf]

Photosynthetic rate

(μmol CO_2 m^{-2} s^{-1})

Treatment		repeat1	repeat2	repeat3	repeat4
18-hour light	NM	3.1825	3.395	3.9775	1.9225
	F.m 20	8.6575	6.5325	9.0575	8.0425
	F.m 50	6.5925	6.56	7.085	6.96
	F.m 200	5.3925	7.2975	7.035	6.5425
6-hour light	NM	2.5675	2.855	3.31	2.5425
	F.m 20	4.73	4.995	4.75	4.75
	F.m 50	4.08	3.68	2.93	4.06
	F.m 200	5.55	4.4075	4.3525	3.43

Transpiration rate

(mmol H_2O m^{-2} s^{-1})

Treatment		repeat1	repeat2	repeat3	repeat4
18-hour light	NM	0.050825	0.06365	0.06595	0.049225
	F.m 20	0.074075	0.076575	0.05475	0.106375
	F.m 50	0.10395	0.105775	0.098325	0.1065
	F.m 200	0.0775	0.089875	0.098175	0.098
6-hour light	NM	0.97675	0.98725	1.255	1.43675
	F.m 20	2.02	2.23	1.365	1.295225
	F.m 50	1.3425	1.5375	1.0025	1.46625
	F.m 200	1.95	2.0155	1.9175	1.34525

Stomatal conductance

(mol H_2O m^{-2} s^{-1})

Treatment		repeat1	repeat2	repeat3	repeat4
18-hour light	NM	0.050825	0.06365	0.06595	0.049225
	F.m 20	0.074075	0.076575	0.05475	0.106375
	F.m 50	0.10395	0.105775	0.098325	0.1065
	F.m 200	0.0775	0.089875	0.098175	0.098
6-hour light	NM	0.0361	0.033975	0.0438	0.04275
	F.m 20	0.125	0.1475	0.103675	0.061025
	F.m 50	0.049925	0.063325	0.0345	0.053925
	F.m 200	0.06795	0.062775	0.072675	0.036825

Reducing sugar content

(mg g^{-1})

Treatment		repeat1	repeat2	repeat3	repeat4
18-hour light	NM	2.249893	2.098733	1.766998	2.397983
	F.m 20	5.115325	4.01405	5.186255	5.518576
	F.m 50	4.841812	4.237365	3.951616	3.216672
	F.m 200	5.938283	6.383704	4.527281	5.059223
6-hour light	NM	0.63024	0.778729	0.832517	0.585907
	F.m 20	5.166333	4.002753	4.4482	4.567633
	F.m 50	4.539494	2.442014	3.368561	4.216524
	F.m 200	4.026104	3.217224	3.014784	3.668215

Starch content

(mg g^{-1})

Treatment		repeat1	repeat2	repeat3	repeat4
18-hour light	NM	3.156327	4.430645	4.555393	3.895992
	F.m 20	5.420829	5.788524	5.469817	5.366237

	F.m 50	5.622372	5.80706	5.860276	7.096268
	F.m 200	8.541551	6.509045	7.547546	6.982162
6-hour light	NM	3.331916	3.766842	2.874261	2.716651
	F.m 20	3.565477	4.606493	3.919308	4.530891
	F.m 50	6.294976	5.614961	5.958187	5.754153
	F.m 200	6.749915	6.628707	7.607356	7.873622

Total soluble sugar content ($mg\ g^{-1}$)

Treatment		repeat1	repeat2	repeat3	repeat4
18-hour light	NM	15.50351	15.47638	13.19715	15.74959
	F.m 20	19.92767	20.31562	23.19787	20.92628
	F.m 50	20.43042	19.27981	18.15878	19.94792
	F.m 200	15.07144	15.96263	15.33986	15.94329
6-hour light	NM	12.62987	11.8651	13.37025	12.39882
	F.m 20	14.28484	14.93513	17.26138	17.33687
	F.m 50	24.49953	20.00343	23.28307	20.45167
	F.m 200	14.92475	14.74047	13.39932	17.25196
