# Supplementary material for: Influence of nutrient signals and carbon allocation on the expression of phosphate and nitrogen transporter genes in winter wheat (Triticum aestivum L.) roots colonized by arbuscular mycorrhizal fungi
Source: PLoS One. 2017 Feb 16;12(2):e0172154. doi: 10.1371/journal.pone.0172154 (PMC5312871; doi:10.1371/journal.pone.0172154)
Supplement: S4 Appendix — (PDF) [file pone.0172154.s004.pdf]

Normalized gene expression

treatment	TaPT4	TaPHT1.2	TaNRT1.1	TaNRT1.2	TaNRT2.1	TaNRT2.2	TaNRT2.3	TaAMT1.2
NM	0.2017	0.1387	0.2192	0.5586	0.0571	0.0173	0.0467	0.0981
NM	0.5743	0.1843	0.1948		0.1921	0.0552	0.0941	0.1615
NM	0.4263	0.0548	0.2483	0.4353	0.2285	0.0529	0.0884	0.1649
NM	0.2912		0.2517	0.3015	0.0556			
F.m-nutrient	0.2698	0.0415	0.2146	0.2382	0.0494	0.0494	0.0321	0.0308
F.m-nutrient	0.5285	0.0608	0.1948	0.1528	0.0203	0.0118	9.16E-03	0.1397
F.m-nutrient	0.054	0.0247	0.5471	0.1638	0.0522	0.0418	0.0111	0.0902
F.m-nutrient	0.0728		0.1843	0.3536	0.0372	0.0181	0.0177	0.1015
F.m+ NO3-	0.1368	0.0208	0.2349	0.2774	0.0775	0.0265	0.0407	0.0647
F.m+ NO3-	0.2415	0.0195		0.1233	0.0464	5.64E-03	6.80E-03	0.0813
F.m+ NO3-	0.2606	0.0135	0.2432	0.2892	0.0579	0.017	0.0286	0.0134
F.m+ NO3-	0.2003		0.2031	0.2253	0.0451	0.0116	4.55E-03	0.012
F.m+NH4+	0.5212	0.0439	0.2755	0.3978	0.0202	0.0127	9.04E-03	0.1111
F.m+NH4+	0.722	0.0347	0.2774	0.2952		0.0212	0.0282	0.1241
F.m+NH4+	0.3035	0.0132	0.1661	0.3463	0.0252	0.0122	0.012	0.1349
F.m+NH4+	0.2643		0.1368	0.2073	0.0166	8.97E-03	3.99E-03	0.1183
F.m+Pi	0.1397	0.0227	0.507	0.5141	0.0458	0.0296		0.0797
F.m+Pi	0.1285	0.0878	0.1241	0.1756	0.0764	8.03E-03	3.00E-03	
F.m+Pi	0.2755	0.0407	0.2892	0.5864	0.0298	0.0198	7.09E-03	0.0634
F.m+Pi	0.067		0.1615	0.2349	0.0116	0.0115	4.04E-03	0.031
