# Supplementary material for: Influence of nutrient signals and carbon allocation on the expression of phosphate and nitrogen transporter genes in winter wheat (Triticum aestivum L.) roots colonized by arbuscular mycorrhizal fungi
Source: PLoS One. 2017 Feb 16;12(2):e0172154. doi: 10.1371/journal.pone.0172154 (PMC5312871; doi:10.1371/journal.pone.0172154)
Supplement: S5 Appendix — (PDF) [file pone.0172154.s005.pdf]

Normalized gene expression

TaNRT1.1	NM	F.M20	F.M50	F.M200
**18-h light**	1.0347	0.2608	0.1965	0.3333
18-h light	0.586	0.2731		0.2521
18-h light	0.285	0.3601	0.2338	
18-h light	0.3956	0.594	0.1767	0.2827
6-h light	0.424	0.1281		0.2001
6-h light	0.431		0.2865	0.293
6-h light	0.5702	0.3652	0.2275	
6-h light	0.6599	0.1238	0.582	

TaNRT1.2	NM	F.M20	F.M50	F.M200
**18-h light**	0.8006	0.5004	0.9878	0.7344
18-h light	0.8699	0.5853	0.4146	0.7131
18-h light	0.6885	0.799	0.4455	0.5481
18-h light	0.7231		0.432	0.7357
6-h light	1.4766	0.2633	1.005	0.7468
6-h light	1.4496	0.2137	0.7405	0.8117
6-h light	0.9135	0.7828	0.8911	1.3156
6-h light	2.1439	0.4023	1.904	0.8766

TaNRT2.1	NM	F.M20	F.M50	F.M200
**18-h light**	0.6594	0.4605	0.6794	0.2726
18-h light	1.2649	0.3578		0.1722
18-h light	0.3168	0.5057	0.2243	0.4271
18-h light	0.3304	0.8115	0.1358	0.482
6-h light	0.5557	0.3287	0.6273	0.3162
6-h light	0.269	0.2122	0.3626	0.4173
6-h light	0.9927	0.3092	0.2339	
6-h light	0.4634	0.1813	0.332	

TaNRT2.2	NM	F.M20	F.M50	F.M200
**18-h light**	0.4861	0.3663	0.3666	0.4428
18-h light	1.601	0.5461		0.304
18-h light	0.2976	0.4817	0.1748	0.6124
18-h light	0.1448	1.0271	0.586	
6-h light	1.1426	0.5644	0.3408	0.5317
6-h light		0.4215	0.6314	0.6194
6-h light	0.8406	0.2529	0.2036	
6-h light		0.3941	0.3229	0.727

TaNRT2.3	NM	F.M20	F.M50	F.M200
18-h light	3.31E-03	3.72E-03	0.0119	7.06E-03
18-h light		5.18E-03	0	2.75E-03
18-h light	3.70E-03	2.10E-03	2.81E-03	3.94E-03
18-h light	1.76E-03	7.64E-03	1.33E-03	0
6-h light	0.0103	5.00E-03		4.48E-03

6-h light	8.42E-04	5.77E-03	4.21E-03	4.45E-03
6-h light	7.97E-03	1.66E-03	3.43E-03	0
6-h light	1.03E-03	1.86E-03	0	0

**TaAMT2.1**	**NM**	**F.M20**	**F.M50**	**F.M200**
18-h light	8.10E-03	1.70E-03	5.36E-04	1.37E-03
18-h light	2.30E-03	1.81E-03		8.90E-04
18-h light	1.16E-03	1.51E-03	3.02E-04	6.72E-04
18-h light	2.29E-03	9.95E-04	5.03E-04	2.89E-03
6-h light	8.65E-04	1.26E-03	1.34E-03	6.30E-04
6-h light	1.11E-03	1.28E-03	1.69E-03	5.30E-04
6-h light	1.39E-03	2.10E-03	8.74E-04	
6-h light	3.88E-03	1.56E-03	8.98E-04	3.01E-04

**TaPT4**	**NM**	**F.M20**	**F.M50**	**F.M200**
18-h light	2.73E+00	7.44E-01	1.15E+00	5.51E-01
18-h light	2.44E+00	5.80E-01	0.054772811	3.40E-01
18-h light	1.33E+00	6.05E-01	8.38E-01	5.06E-01
18-h light	1.63E+00	1.58E+00	4.27E-01	7.04E-01
6-h light	9.12E-01	4.48E-01	1.63E+00	3.33E-01
6-h light	1.12E+00	4.82E-01	5.08E-01	6.73E-01
6-h light	2.40E+00		7.24E-01	0.135939
6-h light	1.44E+00	8.18E-01	1.08E+00	7.41E-01

**TaPHT1.2**	NM	F.M20	F.M50	F.M200
18-h light	1.890734	0.053835	0.090237448	0.043626
18-h light	2.421663	0.109988		0.040402
18-h light	2.590585	0.108339	0.074229495	0.084951
18-h light	1.579011	0.147123	0.033223192	0.22852
6-h light	0.181232	0.047771	0.170938361	0.12373
6-h light	1.867747	0.092604	0.08395121	0.142071
6-h light	2.339926	0.403414	0.106306937	
6-h light	3.220746	0.045117	0.077346298	
